# The Paediatric Admission Quality of Care (PAQC) score: designing a tool to measure the quality of early inpatient paediatric care in a low‐income setting

**DOI:** 10.1111/tmi.12752

**Published:** 2016-08-10

**Authors:** Charles Opondo, Elizabeth Allen, Jim Todd, Mike English

**Affiliations:** ^1^ Health Services UnitKEMRI Wellcome Trust Research ProgrammeNairobiKenya; ^2^ Department of Medical StatisticsLondon School of Hygiene & Tropical MedicineLondonUK; ^3^ Department of Population HealthLondon School of Hygiene & Tropical MedicineLondonUK; ^4^ Department of Epidemiology and BiostatisticsKilimanjaro Christian Medical University CollegeMoshiTanzania; ^5^ Centre for Tropical Medicine and Global HealthNuffield Department of MedicineUniversity of OxfordOxfordUK

**Keywords:** quality of care, hospital, paediatric, score, measure, guideline, qualité des soins, hôpital, pédiatrie, score, mesure, directive

## Abstract

**Background:**

Evaluating clinician compliance with recommended steps in clinical guidelines provides one measure of quality of process of care but can result in a multiplicity of indicators across illnesses, making it problematic to produce any summative picture of process quality, information that may be most useful to policy‐makers and managers.

**Objective:**

We set out to develop a clinically logical summative measure of the quality of care provided to children admitted to hospital in Kenya spanning the three diagnoses present in 60% or more of admissions that would provide a patient‐level measure of quality of care in the face of comorbidity.

**Methods:**

We developed a conceptual model of care based on three domains: assessment, diagnosis and treatment of illnesses. Individual items within domains correspond to recommended processes of care within national clinical practice guidelines. Summative scores were created to reduce redundancy and enable aggregation across illnesses while maintaining a clear link to clinical domains and our conceptual model. The potential application of the score was explored using data from more than 12 000 children from eight hospitals included in a prior intervention study in Kenya.

**Results:**

Summative scores obtained from items representing discrete clinical decision points reduced redundancy, aided balance of score contribution across domains and enabled direct comparison of disease‐specific scores and the calculation of scores for children with comorbidity.

**Conclusion:**

This work describes the development of a summative Paediatric Admission Quality of Care score measured at the patient level that spans three common diseases. The score may be an efficient tool for assessing quality with an ability to adjust for case mix or other patient‐level factors if needed. The score principles may have applicability to multiple illnesses and settings. Future analysis will be needed to validate the score.

## Abstract (fr)

**Contexte:**

L’évaluation de l'adhésion des cliniciens aux étapes recommandées dans les directives cliniques fournit une mesure de la qualité du processus des soins, mais peut conduire à une multiplicité d'indicateurs à travers les maladies, ce qui rend problématique la production d'une image sommative de la qualité des processus, des informations qui peuvent être plus utiles pour les décideurs et les gestionnaires.

**Objectif:**

Nous avons cherché à élaborer une mesure sommative cliniquement logique de la qualité des soins dispensés aux enfants admis à l'hôpital au Kenya, couvrant les trois diagnostics posés dans 60% ou plus des admissions, qui pourrait fournir une mesure à l’échelle du patient de la qualité des soins face à la comorbidité.

**Méthodes:**

Nous avons développé un modèle conceptuel de soins basé sur trois domaines: l’évaluation, le diagnostic et le traitement des maladies. Les éléments individuels au sein des domaines correspondent à des processus de soins recommandés dans les directives nationales de la pratique clinique. Les scores sommatifs ont été créés pour réduire la redondance et permettre l'agrégation des maladies, tout en maintenant un lien clair avec les domaines cliniques et notre modèle conceptuel. L'application potentielle du score a été explorée à l'aide des données de plus de 12.000 enfants de 8 hôpitaux inclus dans une étude d'intervention préalable au Kenya.

**Résultats:**

Les scores sommatifs obtenus à partir d’éléments représentant des points de décision clinique discrète ont réduit la redondance, aidé à l’équilibrage de la contribution des scores entre les domaines et permis une comparaison directe de scores spécifiques à la maladie et le calcul de scores pour les enfants avec des comorbidités.

**Conclusion:**

Ce travail décrit le développement d'un score sommatif de la qualité des soins d'admission pédiatrie (QSAP) mesuré à l’échelle du patient qui couvre 3 maladies courantes. Le score peut être un outil efficace pour évaluer la qualité, avec une capacité d'ajustement pour des cas mixtes ou pour d'autres facteurs à l’échelle du patient si nécessaire. Les principes du score peuvent avoir une applicabilité à plusieurs maladies et cadres. Des analyses ultérieures seront nécessaires pour valider le score.

## Abstract (es)

**Antecedentes:**

La evaluación del cumplimiento clínico de los pasos recomendados en las guías clínicas provee una medida de la calidad de los procesos de atención sanitaria pero puede resultar en una multiplicidad de indicadores del proceso de enfermedad, siendo problemático producir cualquier foto completa de la calidad del proceso, información que podría ser sobretodo útil para los políticos y gestores.

**Objetivo:**

Nos propusimos desarrollar una medida clínica agregada lógica de la calidad de atención brindada a niños admitidos al hospital en Kenia comprendiendo los tres diagnósticos presentes en un 60% o más de las admisiones, que proveerían una medida a nivel del paciente de la calidad de los cuidados frente a la comorbilidad.

**Métodos:**

Hemos desarrollado un modelo conceptual de atención sanitaria basado en tres dominios: evaluación, diagnóstico y tratamiento de la enfermedad. Ítems individuales dentro de los dominios corresponden a procesos de atención sanitaria recomendados dentro de las guías clínicas nacionales. Se crearon puntajes agregados para reducir la redundancia y permitir la agregación de enfermedades a la vez que se mantiene un nexo claro con los dominios clínicos y nuestro modelo conceptual. La aplicación conceptual de la puntuación se exploró utilizando datos de más de 12,000 niños de 8 hospitales incluyendo un estudio de intervención previo en Kenia.

**Resultados:**

Las puntuaciones aditivas obtenidas para los ítems que representan puntos discretos de decisión clínica redujeron la redundancia, ayudaron a balancear la contribución de las puntuaciones a lo largo de los dominios y permitieron una comparación directa de puntuaciones específicas de una enfermedad y el cálculo de puntuaciones para niños con comorbilidades.

**Conclusión:**

Este trabajo describe el desarrollo de una puntuación aditiva de la calidad en la atención en admisión pediátrica (PAQC) medida a nivel del paciente y que incluye tres enfermedades comunes. La puntuación podría ser una herramienta eficiente para evaluar la calidad, con posibilidad de ajustar a casos mixtos u otros factores a nivel del paciente si fuese necesario. Los principios de la puntuación podrían aplicarse a enfermedades y lugares múltiples. Se requieren análisis futuros para validar la puntuación.

## Introduction

Measuring quality of health care is an important aspect of any health system as it provides the information necessary to monitor and improve service delivery. However, quality of care is a multifaceted concept 1, 2, 3 so it is important to deconstruct ‘quality’ to enable measurement and allow for a clearer understanding of its components. Such thinking led to the most commonly discussed framework for measuring quality proposed by Avedis Donabedian when he described three attributes of quality of care, namely structures, processes and outcomes 4. Processes refer to what is actually done by health workers in providing care, such as taking a clinical history, performing a physical examination, making a diagnosis and initiating treatment to restore health. While many indicators based on specific components of clinicians’ practice have been reported, there is currently no tool that allows aggregation across common care tasks at admission to hospital for sick children. Here, we describe development of a tool to do this, the Paediatric Admission Quality of Care (PAQC) score. Although this is developed for African settings, we believe the principles of score development are more widely applicable.

We use recommendations summarised in evidence‐based clinical practice guidelines as standards of technical quality of clinical processes including clinical assessment and treatment. This is a widely adopted approach to assessing process quality 5, 6 with simulations 7, vignettes 8 or review of practice records 9, 10 to obtain the requisite data. Typically, these yield large numbers of results based on itemised components of the clinical process (e.g. whether a specific clinical sign was evaluated) or aggregate measures (e.g. a percentage score across a set of steps). Yet different audiences may have different preferences for reports on quality. For example, a clinical team leader may wish to know which specific clinical steps are poorly (or well) performed. A programme manager may wish to know the overall quality of care for a specific disease, while a policy‐maker may simply wish to see a single result representing how good or bad things are whatever the disease. The first requirement demands measures that retain granularity – details of the quality of care for each action. The second requires a summary across all measures associated with a disease or illness, and the third requires a summary across multiple conditions (or diseases), potentially even in the same patient in the occurrence of comorbidity. We aimed to develop aggregate measures that retain granularity that might be used to examine whether quality is changing over time or across health facilities. Our initial focus was developing a score that summarises the quality of clinical care provided to children with malaria, pneumonia and diarrhoea/dehydration on admission to hospital, because these three illnesses are responsible for over 60% of hospital admissions and deaths in children aged 1–59 months globally 11, 12.

## Methods

### Conceptual model of care according to guidelines

**Figure 1:**
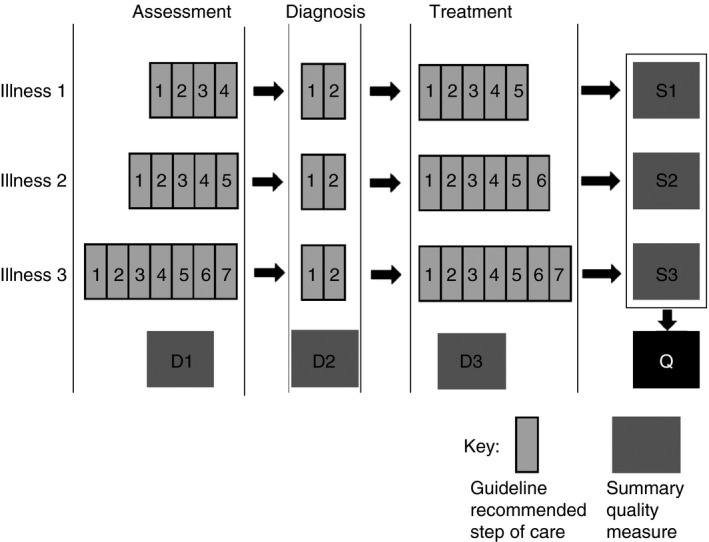
Outline of the proposed measure showing various levels of summary up to the individual level. Items contributing to the measure and which may be unique for each illness are labelled 1 through 7 while D1 through D3 represent domain‐level aggregate measures for each illness or combination of illnesses. S1 through S3 are summary measures for each illness. Q is the overall measure at the individual level, which when averaged for children attended to by the same clinician or at a department or hospital constitute higher‐level aggregate measures.

In Kenya, standards of inpatient admission care for children are defined in a set of practice guidelines for healthcare workers 13. From these protocols, three distinct domains in the process of care can be defined which not only represent different dimensions of process but also distinct competencies. An initial assessment domain encompasses the documentation of signs and symptoms. This domain is followed by the diagnosis phase in which clinical information is integrated, a process supported by clinical algorithms in the written guidelines. In the third domain, treatment should be accurately prescribed based on the diagnosed illness(es) and their severity classification. Conceptually, therefore the process of care – and thus component indicators – can be divided into these three domains for any illness episode (Figure 1).

Aggregating component indicator scores across domains might provide a score for an individual illness episode allowing calculation of a summarised illness score for multiple similar episodes. Aggregating across illnesses might create scores that allow summaries within an individual or for a set of individuals with multiple diagnoses. However, if simply adding itemised steps for different illnesses results in different total scores for specific illnesses then their scores are not directly or intuitively comparable, and creation of a patient‐specific score in the face of comorbidity becomes problematic. Here, we illustrate the problem of using simple additive scores based on all items and propose the PAQC score as a solution to this problem.

### Data used to explore and develop the PAQC score

The data for designing and testing the PAQC score came from a previously published large, pragmatic trial of a multifaceted intervention to improve quality of care for children 14, 15. This dataset of 12 036 children admitted with acute illnesses to 8 Kenyan district hospitals includes baseline observations, before any intervention and post‐intervention observations (Table S1). The effect of intervention on specific, pre‐specified process indicators has previously been reported 14. Methods for collecting these data have been described in detail elsewhere, but in brief, they were collected using a patient case‐record data abstraction form by data collectors trained for 3 weeks and sent to the study sites in four teams each supervised by a research team member. Each team, made up of four individuals, abstracted the data from approximately 400 case records in each hospital over 6–7 days. Duplicate collection of 10% of the data at each site during each survey was undertaken to assess agreement which was consistently found to be above 95%. Continued experience with this method of data collection, including comparisons of retrospective and prospective data collection 16, point to its feasibility and value for assessing specific items of quality of care in paediatric 17, neonatal 10 and surgical 18 care with potential to integrate this into longer‐term data collection systems 19 and potentially even electronic health records.

### A basic additive score

Component indicators (items) correspond to specific recommendations on the process of care in the guidelines (Table 1). They are binary items, scored 1 if undertaken as recommended and 0 otherwise, within the three domains: assessment, diagnosis and treatment.

**Table 1:** Items in the domains of the basic score

	Malaria	Pneumonia	Diarrhoea/dehydration
**Assessment** Each item scored 1 if documented (present, absent, quality or quantity) and 0 otherwise	Fever Convulsions Acidotic breathing Pallor (In)ability to drink or breastfeed Level of consciousness (AVPU) Indrawing Blood test for malaria	Cough Difficult breathing Central cyanosis (In)ability to drink or breastfeed Level of consciousness (AVPU) Grunting Indrawing Respiratory rate	Diarrhoea Vomiting Capillary refill (In)ability to drink or breastfeed Level of consciousness (AVPU) Sunken eyes Return of skin pinch Character of pulse
**Diagnosis** Item score is 1 if a relevant severity classification is indicated, 0 otherwise	Classification: severe or non‐severe	Classification: very severe, severe or non‐severe	Classification: shock, severe, some or none
**Treatment** Score items depend on severity classification ‘Drug’ score is 1 if correct (singly or in recommended combinations where applicable) according to guidelines for indicated severity classification ‘Route’, ‘dose’, ‘duration’ and ‘frequency’ each score 1 if correct (singly and in combination where applicable) for choice of drug(s) according to guideline recommendations for their use, 0 otherwise	Severe malaria: Drug: quinine (loading and maintenance) Route: IV or IM Dose: 20 mg/kg loading, 10 mg/kg maintenance ±20% Frequency: twice daily Duration: Stat for loading dose and any duration for maintenance dose Non‐severe malaria: Drug: artemether‐lumefantrine or quinine Route: oral Dose: 5–14.9 kg – 1 tab; 15–24.5 kg – 2 tabs; 25–34.9 kg – 3 tabs; 35 kg+ – 4 tabs Frequency: twice daily for AL and thrice daily for quinine Duration: any duration specified	Very severe pneumonia: Drug: penicillin and gentamicin and oxygen Route: IV or IM Dose: Penicillin 50 000 IU/kg, gentamicin 7.5 mg/kg (both ± 20%) Frequency: Penicillin ×4, Gentamicin ×1, oxygen any specified Duration: any specified Severe pneumonia: Drug: Penicillin only (no gentamicin) Route: IV or IM Dose: 50 000 IU/kg ±20% Frequency: ×4 Duration: any specified Non‐severe pneumonia: Drug: Amoxicillin or cotrimoxazole Route: oral Dose: Amoxicillin 25 mg/kg, cotrimoxazole 24 mg/kg ±20% Frequency: Amoxicillin ×3, cotrimoxazole ×2 Duration: any specified	Shock: Drug: normal saline or Ringer's lactate/Hartmann's solution Dose: volume/time ×4 within ±20% of 20 ml/kg Frequency: at least 1 in an hour Severe dehydration: Drug: Ringer's or ORS Dose: total vol/time within ±20% of 30 ml/kg + 70 mg/kg in 3 h for >1 year or in 6 h for <1 year of Ringer's or total vol/time within ±20% of 100 ml/kg in 6 h. Frequency: step 1/2 used Some dehydration: Drug: ORS Dose: vol/time ×4 within ±20% of 75 ml/kg Frequency: at least 1 in an 24 h No dehydration: Drug: ORS Dose: 10 ml/kg ±20% Frequency: any specified

The assessment domain score was the number of signs and symptoms documented by the admitting clinician. The diagnosis domain score was a binary indicator of whether the clinician made a valid classification of the severity of illness recognised in the guidelines. For the treatment domain, indicators based on recommendations on dosages, route and frequency, and durations of treatment were summed up. Deviations of up to 20% of recommended dosages per kilogram of body weight, which are within therapeutically safe dose ranges for all the drugs used, were considered to be correct.

There were 19 guideline‐recommended signs and symptoms necessary for identifying and classifying the severity of the three illnesses we focused on. Two of these – ability to drink or breastfeed and level of consciousness – were common across all three diseases. Five treatment indicators were defined to score the treatment of malaria and pneumonia but only three of these – treatment choice, dose and frequency – were applicable to diarrhoea/dehydration. A basic score was created as an arithmetic sum of each item in the assessment, diagnosis and treatment domains. However, a number of problems were observed with the characteristics of this score: they include redundancy between items within domains, domination of the scale by items from one domain (assessment), and non‐equivalent scores across diseases. For these reasons, this approach to creating scores was rejected.

### Disease‐specific PAQC scores

To overcome the problems of the simple additive scores, we collapsed all of the original items within domains into new components representing discrete clinical decision points that constitute the desired processes outlined in the guidelines. The resulting domain‐specific components, listed in Table 2, were designed with the aim of making them generic to the process of care of all three diseases and arguably most other acute childhood illnesses. To this end, assessment was defined by three components: (i) primary assessment signs required to diagnose the disease of interest; (ii) secondary assessment signs necessary to distinguish between disease severity classifications; and (iii) a third item representing complete documentation of all required assessment signs.

**Table 2:** Items in the domains of the PAQC score

Domain	Disease		
	Malaria	Pneumonia	Diarrhoea/dehydration
**Assessment** Each grouped item scored 1 if all of its elements are documented (present, absent, quality or quantity) and 0 otherwise	Primary signs: fever Secondary signs: convulsions or acidotic breathing or (in)ability to drink/breastfeed or AVPU, or pallor in the presence of grunting or indrawing if severe, or convulsions and acidotic breathing and (in)ability to drink/breastfeed or AVPU, or pallor and grunting and indrawing if non‐severe Complete assessment: all signs documented	Primary signs: cough or difficult breathing Secondary signs: central cyanosis or (in)ability to drink/breastfeed or AVPU or grunting or acidotic breathing if very severe, or central cyanosis and (in)ability to drink/breastfeed or AVPU, and grunting and acidotic breathing if severe, or central cyanosis and (in)ability to drink/breastfeed or AVPU, and grunting and acidotic breathing and respiratory rate if non‐severe. Complete assessment: all signs documented	Primary signs: diarrhoea and/or vomiting Secondary signs: capillary refill or AVPU or (in)ability to drink/breastfeed, and pulse if shock, or capillary refill and AVPU or (in)ability to drink/breastfeed and sunken eyes and skin pinch and pulse if severe, some or no dehydration Complete assessment: all signs documented
**Diagnosis** Item score is 1 if a relevant severity classification is indicated, 0 otherwise	Classification: severe or non‐severe	Classification: very severe, severe or non‐severe	Classification: shock, severe, some or none
**Treatment** ‘Drug’ score is 1 if correct (singly or in recommended combinations where applicable) according to guidelines for indicated severity classification ‘Correct use’ scores 1 if dose, route, frequency and duration whichever applicable, of selected drug(s) are correct following guideline recommendations for their use, 0 otherwise	Severe malaria: Drug: quinine (loading and maintenance) Correct use: Route is IV or IM and dose is 20 mg/kg loading, 10 mg/kg maintenance ±20% and frequency is twice daily and duration is stat for loading dose and any duration for maintenance dose Non‐severe malaria: Drug: artemether‐lumefantrine or quinine Correct use: Route is oral and dose is 5–14.9 kg – 1 tab; 15–24.5 kg – 2 tabs; 25–34.9 kg – 3 tabs; 35 kg+ – 4 tabs, and frequency is twice daily for AL and thrice daily for quinine and duration is any duration specified	Very severe pneumonia: Drug: penicillin and gentamicin and oxygen Correct use: Route is IV or IM and dose is penicillin 50 000 IU/kg, gentamicin 7.5 mg/kg (both ±20%) and frequency is penicillin ×4, gentamicin ×1, oxygen any specified and duration is any specified Severe pneumonia: Drug: Penicillin only (no gentamicin) Correct use: Route is IV or IM and dose is 50 000 IU/kg ±20% and frequency is ×4 and duration is any specified Non‐severe pneumonia: Drug: Amoxicillin or cotrimoxazole Correct use: Route is oral and dose is Amoxicillin 25 mg/kg, cotrimoxazole 24 mg/kg ±20% and frequency is Amoxicillin ×3, cotrimoxazole ×2 and duration is any specified	Shock: Drug: normal saline or Ringer's lactate/Hartmann's solution Correct use: Dose is volume/time×4 within ±20% of 20 ml/kg and frequency is at least 1 in an hour Severe dehydration: Drug: Ringer's or ORS Correct use: Dose is total vol/time within ±20% of 30 ml/kg + 70 mg/kg in 3 h for >1 year or in 6 h for <1 year of Ringer's or total vol/time within ±20% of 100 ml/kg in 6 h and frequency is step 1/2 used Some dehydration: Drug: ORS Correct use: Dose is vol/time ×4 within ±20% of 75 ml/kg and frequency: at least 1 in an 24 h No dehydration: Drug: ORS Correct use: Dose is 10 ml/kg ±20% and frequency is any specified

For example, for malaria, the primary assessment sign was fever. Secondary signs depended on severity. According to guidelines, severe malaria was the correct diagnosis for a child who, in addition to fever, presented with at least one danger sign – convulsion, acidotic breathing, inability to drink or breastfeed, altered consciousness or pallor with respiratory distress indicated by grunting or indrawing. Fever in the absence of any danger sign was to be classified as non‐severe malaria. A clinician was required to completely exclude the presence of danger signs to correctly diagnose non‐severe malaria. For this reason, a complete secondary assessment for non‐severe malaria meant documentation of all the danger signs. The assessment domain score thus rewarded identification of danger signs and completion of all assessment tasks as recommended in the guidelines. This approach was also applied to children diagnosed with pneumonia and diarrhoea/dehydration.

For the diagnosis domain, the binary indicator of whether a relevant severity classification was made was retained unchanged. However for treatment, two domain‐specific components were generated: (i) selection of a relevant drug for treatment of the disease diagnosed and (ii) correct use of the selected drug which included correct dose, appropriate route of delivery, frequency and duration where applicable. The resulting disease‐specific score was a sum of these domain scores for each individual.

### Moving from disease‐specific scores to an overall patient‐level PAQC score

Where a child had only one of our three diagnoses, their disease‐specific score became the patient‐level PAQC score. To measure quality of care in an admission episode with more than one illness, a score combining the disease‐specific scores was needed. An intuitive approach would be to use the arithmetic mean of the disease‐specific scores. However, this approach would have created non‐integer score values which no longer represented a count of guideline‐recommended process‐of‐care tasks completed by the clinician. Thus, an alternative approach was used in which a domain‐specific score was 1 if the equivalent items in each of the diagnosed diseases had scored 1 and zero otherwise – an all‐or‐none combination of disease‐specific, domain‐specific scores. For example if a child had malaria and pneumonia, then the primary assessment score was 1 if primary assessment items for both malaria and pneumonia (presence of fever documented, and presence of cough or difficult breathing documented) scored 1; if only one or none of them were documented then the item score was zero. Although this approach made it more difficult to achieve each level of the score, it reflected the clinical reality that multiple diagnoses increase the number of guideline‐recommended tasks required to effectively manage illness thereby increasing the difficulty in providing quality care when there is multimorbidity.

In sum therefore, at the patient level, the PAQC score is represented by the disease‐specific score where there is a single diagnosis and by a score that incorporates multimorbidity when the child has more than one diagnosis. It remains possible to report these disease‐specific scores (or scores within their domains) if there is a specific interest in these as outcomes or for monitoring and improvement management purposes. However, it is also now possible to report a patient‐level PAQC score that spans all three diseases and combinations of these diseases.

### Testing score properties

As well as basing score construction on a logical clinical strategy, we also examined score properties. Tetrachoric correlation coefficients were used to flag pairs of items within the same domain that were similar enough to be deemed redundant 20. Correlations greater than 0.80 are considered to be ‘very strong’ according to the criteria suggested by Evans 21 indicating such items might need to be removed or combined with others in the same domain where it was clinically meaningful to do so. Internal consistency and face validity of the diseases‐specific scores and patient‐level PAQC score was explored by checking that score differences between groups and across time were consistent with the improvement in quality‐of‐care indicators that has been documented previously 14. An ordinal hierarchical regression model allowing for clustering of observations within hospitals was used to explore improvement in quality as measured by the PAQC score. This model adjusted for multimorbidity in the light of its potential to complicate care.

## Results

A simple additive approach across the domains assessment, diagnosis and treatment resulted in a 15‐point score (range 0–14) for malaria and pneumonia and a 13‐point score (range 0–12) for diarrhoea/dehydration. Each had a 9‐point (range 0–8) score for the assessment domain, a 2‐point (binary) score for the diagnosis domain but a 6‐point (range 0–5) score for the treatment domain for malaria and pneumonia and a 4‐point (range 0–3) score for diarrhoea/dehydration. Thus, these simple additive scores were heavily weighted towards the assessment domain. The differences in scale ranges across diseases (0–14 for malaria and pneumonia, 0–12 for diarrhoea/dehydration) also made comparisons of quality of care across diseases less intuitive and presented problems when reporting an overall quality score for a patient with multiple morbidity. Furthermore, there was a high degree of correlation between multiple items in this simple additive score as shown by the tetrachoric correlation coefficients (Tables S2 and S3). Additionally, the malaria assessment and treatment item tetrachoric correlation matrices were not positive (semi)definite, suggesting a high degree of linear dependency between multiple items. These findings imply considerable redundancy and are a justification for rejecting these simple additive scores.

For the disease‐specific scores, tetrachoric correlation coefficients of within‐domain components of the score ranged between 0.34 and 0.62 – ‘weak’ to ‘moderate’ according to Evans’ criteria – showing much less of the codependence observed between items in the simple additive score (Table S4). Between‐domain correlation of items was also in this range. However, there was a perfect correlation between the classification indicator and the drug choice indicator in malaria and pneumonia; this was expected because the choice of drug depended on severity classification and for this reason neither indicator was dropped. There is therefore support for retaining the disease‐specific scores as a 7‐point score (range 0–6) across all three diseases, being the sum of six binary items (Table 2) contributing to it. These items were grouped in the assessment domain (range 0–3 points), diagnosis (range 0–1 point) and the treatment domain (range 0–2 points). Our approach to building a score for children with multiple diseases retains this points system.

Using data from the intervention study, we examined the proportion of children with specific diseases or with multimorbidity for whom each of the six binary items was scored 1 at baseline and endline (Table 3). Performance across items varied widely: assessment of signs of disease was poor at baseline with none of the children having a complete assessment across the three diseases, although this had improved to between 22.7% and 43.3% at the endline survey. At the other end of the spectrum documentation of primary signs of illness was performed for over 80% of children at baseline and over 90% at the endline survey for almost all disease‐specific and multimorbidity scores. Performance tended to be lower in children with multimorbidity than in those with one disease.

**Table 3:** Percentages of children with single and multimorbidity for whom items were scored 1 across all hospitals in the baseline and endline surveys

Items	Disease‐specific PAQC scores						Multimorbidity PAQC scores								Overall patient‐level PAQC score	
	Malaria		Pneumonia		Diarrhoea/Dehydration		Malaria + Pneumonia		Malaria + Diarrhoea		Pneumonia + Diarrhoea		Malaria + Pneumonia + Diarrhoea			
	Baseline *n* = 953	Endline *n* = 750	Baseline *n* = 304	Endline *n* = 419	Baseline *n* = 108	Endline *n* = 208	Baseline *n* = 548	Endline *n* = 579	Baseline *n* = 225	Endline *n* = 328	Baseline *n* = 13	Endline *n* = 64	Baseline *n* = 37	Endline *n* = 132	Baseline *n* = 2188	Endline *n* = 2480
Primary signs/symptoms of illness documented	88.56	93.87	92.11	99.05	99.07	98.08	85.77	95.34	73.33	93.90	92.31	93.75	62.16	96.97	86.88	95.60
Secondary signs/symptoms of illness documented	38.51	75.73	0.00	70.88	0.00	35.58	0.18	56.99	0.00	27.44	0.00	26.56	0.00	20.45	16.82	56.57
Complete documentation of signs/symptoms	0.00	40.53	0.00	42.00	0.00	43.27	0.00	34.20	0.00	30.49	0.00	32.81	0.00	22.73	0.00	37.06
Illness severity classification made consistent with guidelines	9.86	57.60	15.13	85.68	43.52	70.67	0.73	64.25	0.00	48.78	0.00	60.94	0.00	52.27	8.73	63.63
Choice of drug for treatment is consistent with illness severity as recommended in guidelines	8.29	40.80	1.32	20.53	27.78	64.42	0.00	12.61	0.44	26.52	0.00	25.00	0.00	7.58	5.21	28.71
Selected drug used as recommended in guidelines	0.10	31.47	32.57	56.80	25.00	46.63	0.00	18.83	0.00	17.68	7.69	35.94	0.00	4.55	5.85	30.93
Score median (IQR)	1 (1–2)	4 (2–5)	1 (1–2)	4 (3–5)	2 (1–2)	4 (2–5)	1 (1–1)	3 (2–4)	1 (0–1)	2 (1–4)	1 (1–1)	2 (2–4)	1 (0–1)	2 (1–3)	1 (1–2)	3 (2–4)

**Figure 2:**
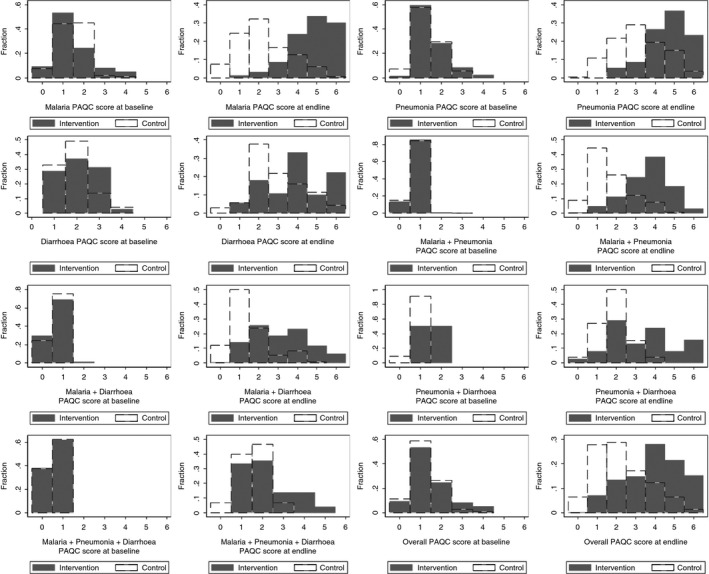
Distributions of the disease‐specific, multimorbidity and overall PAQC score comparing baseline and main endline scores in the intervention and control hospitals.

The overall PAQC score mirrored the trends observed with disease‐specific and multimorbidity scores, ranging from an 8.7% increase in the documentation of primary signs to a 54.9% improvement in the documentation of illness severity classifications between the two surveys. Furthermore, the distribution of the PAQC scores from baseline through to the post‐intervention survey was also shifted towards higher scores in the intervention group as shown in Figure 2. In the multimorbidity adjusted hierarchical ordinal regression analysis, the proportional odds for higher PAQC scores in the intervention and control groups were similar at baseline (pOR 0.86, 95% CI 0.45–1.64, *P*‐value 0.640). Although the control group had higher PAQC scores in successive surveys (pOR 1.58, 95% CI 1.51–1.66, *P*‐value <0.001), the intervention group showed a bigger increase (group–survey interaction pOR 2.00, 95% CI 1.87–2.14, *P*‐value < 0.001). These findings are consistent with previously documented effects of the intervention 14 and are therefore a testament to the internal consistency of the PAQC score. It was possible to include 2188 and 2480 children in the analysis of scores at baseline and endline, respectively, when using the PAQC score *vs*. between 13 and 953 children with disease‐specific and multimorbidity scores. This illustrates the potential gain in efficiency that may result from having a PAQC score that provides a patient‐level measure of quality for multiple diagnoses.

## Discussion

We have described the development of the Paediatric Admission Quality of Care (PAQC) score to measure the processes of admission care for children with the three commonest diseases resulting in hospitalisation and death. The approach aggregates items representing processes of care recommended in established practice guidelines into a single metric, and items are further grouped into domains representing discrete dimensions of process of care. To reduce redundancy between items, maintain reliability and validity of the measure and to create generic components capable of measuring process of care for a variety of diseases, we have systematically combined similar indicators within the domains. The score itself focuses on a critical period for treatment of acute illnesses when there is sufficient opportunity to intervene and restore health in low‐income countries 22, 23, 24, 25, 26, 27. We propose use of an overall PAQC score although the procedure for its calculation also allows the calculation of malaria‐, pneumonia‐ and diarrhoea‐specific PAQC component scores. We have demonstrated the sensitivity of the resulting measures to changes in quality of care that have previously been documented 14.

The PAQC score begins to fulfil the need for a well‐developed quality assessment tool relevant for use in a low‐income setting where less than optimal care provided by health workers 5, 6 is often a major limitation to achieving good outcomes 8. The score also addresses a number of difficulties encountered in quality‐of‐care measurement in general. Firstly, deriving the score from generic indicators of process of care instead of disease‐specific items allows for the direct comparison of disease‐specific PAQC component scores. The score can be further decomposed if such granularity is required to allow reporting of domain and disease‐specific scores catering to the needs of different levels of decision‐making. The ability to encompass multiple steps in the process of care defined in guidelines is in contrast to many previous reports of quality‐of‐care measurement in which individual guideline steps are reported as indicators treating each as an independent event.

Box 1 **Suggested steps to creating PAQC scores**

- Step 1: Identify the health facilities to be surveyed and assign them unique identity codes. If possible, identify the clinicians responsible for providing admission care to children and assign them unique identity codes too.
- Step 2: Create a sampling frame of case records to be selected for abstraction. This should include the age range, illnesses of interest, period and number – whether all or a subset – of eligible children whose records will be abstracted.
- Step 3: Obtain the case records of the children corresponding to the sampling frame and assign them unique identity numbers.
- Step 4: Use a patient case‐record data abstraction form (electronic or paper‐based) to obtain the data required to calculate the PAQC score, and import the data into any suitable statistical software.
- Step 5: For each child create binary indicators for each of the disease‐specific and overall PAQC scores. The indicators of what constitutes good care should be based on the most recent guidelines, or published evidence in the absence of guidelines.
- Step 6: Create the disease‐specific and overall PAQC scores for each child by adding up over their disease‐specific and overall PAQC score indicators.
- Step 7: Obtain a suitable average (e.g. mean, median) of the individual (child)‐level PAQC scores at the required level of reporting (e.g. clinician, hospital, ward, e.t.c.).

An advantage of the patient‐level PAQC score is its ability to combine data on quality of care for three common diseases including children with multimorbidity in one summary index. This could make it an efficient endpoint for testing interventions or quality improvement efforts as fewer patients may need to be included in studies. However, the score may be affected by the distribution of diagnoses in the population under study. Patient‐level measurement provides the flexibility to adjust not only for such variations in case mix but also for characteristics such as age, sex, severity of illness and frequency of comorbidity in statistical models. Where data allow it also provides for aggregation at clinician, department and hospital level when contrasting performance across places or time and in response to interventions.

Measuring quality of care in terms of process by contrasting what was documented to have been undertaken – or the lack of documentation – with guideline recommendations is a relatively narrow perspective for measuring what is obviously a multifaceted concept; it is nevertheless a very important one considering the central role of clinical processes in providing the means by which health inputs are converted to desirable outcomes and the transparent link it offers between evidence‐based recommendations and practice. This approach to measurement can potentially be extended to other situations – for example, other diseases in childhood or other clinical fields such as surgical admission –as the principle underlying the three domains of process is almost universal in medicine and can be adapted to accommodate variation in guidelines across place or time. The absence of a fourth domain of items relating to diagnostic testing is a weakness worth noting. Perhaps, this reflects the low‐resource nature of this setting where the use of such technologies is not widespread or emphasised as important for the delivery of care 28. Future work could explore adding this domain in settings where such elements of process are clearly part of the standards of admission care. Validation of the score to demonstrate links between process and an objective outcome of care, such as mortality, would also be a key step in demonstrating the relevance of clinical processes in quality‐of‐care assessment in a low‐income country. The validation process could also explore the utility of the score in routine care setting where quality of care is likely to differ from a trial setting, and its applicability to a variety of health facilities up and down the referral chain. It would involve using new data to replicate the score then applying suitable statistical techniques to investigate its association with relevant outcomes.

## Conclusion

This work shows how a quality‐of‐care score, the PAQC score, aimed at childhood admissions to low‐income hospital settings has been derived to provide a clinically logical summative measure of the process of care for common childhood illnesses. Future work will be required to explore the validity of the score, its potential value as a measure used to test interventions or track changes in quality and its acceptance by and value to health systems managers. The approach taken may be of value for other clinical settings, including non‐paediatric care, non‐medical care and non‐communicable diseases.

## Supporting information


**Appendix S1.** The paediatric data abstraction form.

**Appendix S2.** Anti‐malarial drug doses.

**Appendix S3.** Oral antibiotic doses.

**Appendix S4.** Intravenous/intramuscular antibiotic doses – ages 7 days and older.

**Appendix S5.** Urgent fluid management – child without severe malnutrition.

**Appendix S6.**
**Table S1.** Number of episodes of each disease/multi‐morbidity across surveys.
**Table S2.** Tetrachoric correlation matrix of assessment items in the basic score.
**Table S3.** Tetrachoric correlation matrix of treatment items in the basic score.
**Table S4.** Tetrachoric correlation matrix of items in the malaria, pneumonia and diarrhoea/dehydration component PAQC scores.
