# Supplementary material for: The Paediatric Admission Quality of Care (PAQC) score: designing a tool to measure the quality of early inpatient paediatric care in a low‐income setting
Source: Trop Med Int Health. 2016 Aug 10;21(10):1334–45. doi: 10.1111/tmi.12752 (PMC5053245; doi:10.1111/tmi.12752)
Supplement: Supplementary file 1 — Appendix S1. The paediatric data abstraction form. [file TMI-21-1334-s001.docx]

## The paediatric data abstraction form

| Hosp ID. |  | | | | | HW code | | | | | |  | | | | | | | | Questionnaire No. | | | | | | |  | | | | | IP NO. | | | |  | QID |  | | | |
| --- | --- | --- | --- | --- | --- | --- | --- | --- | --- | --- | --- | --- | --- | --- | --- | --- | --- | --- | --- | --- | --- | --- | --- | --- | --- | --- | --- | --- | --- | --- | --- | --- | --- | --- | --- | --- | --- | --- | --- | --- | --- |
| Name  (First, Last) |  | | | | | | | | | | | | | | | | | | | | | | | | | | Age | | | | | | | | | yrs | | mths | | | |
| Adm Date | / / 20 | | | | | | | | | | Wt (kg) | | | | | | | | |  | | | | | | | Ht (cm) | | | |  | | | | | WHZ | |  | | | |
| Sex | M / F / E | | | | | | | | | | Temp (^0^C) | | | | | | | | |  | | | | | | | Vaccines | | | | *PCV10 X....../E* | | | | | *M’sles*  Y / N / E | | *DTP/ Penta X....../E* | | | |
| Is there a hospital folder with an IP number? | | | | | | | | | | | | | | | | | | | | | | | | | | | | | | | | | | | | | | Y / N | | | |
| History | | | | | | | | | | | | | | | | |  | | | | Examination | | | | | | | | | | | | | | | | | | | | |
| Length of illness | | | | days | | | | | | | | | | | | |  |  |  |  | Airway | | | | | Clear | | | Stridor | | | | | | | Needs active support to open | | | | | E |
| Fever | | | | Y | | | | |  | | | | E | | | |  | | | | Breathing | | | | | Respiratory Rate | | | | | | | | | | /min / E | | | | | |
| Cough | | | | Y | | | | | N | | | | E | | | |  |  |  |  |  | | | | | Oxygen saturation | | | | | | | | | | | _ _ % SpO2 | | | | |
|  | | | |  | | | | |  | | | |  | | | |  |  |  |  |  |  |  |  |  | Central Cyanosis | | | | | | | | | | | Y | N | | E | |
| Cough > 3 weeks | | | | Y | | | | | N | | | | E | | | |  |  |  |  |  |  |  |  |  | Indrawing | | | | | | | | | | | Y | N | | E | |
| Difficulty breathing | | | | Y | | | | | N | | | | E | | | |  |  |  |  |  |  |  |  |  | Grunting | | | | | | | | | | | Y | N | | E | |
|  | | | | | | | | | | | | | | | | |  | | | |  | | | | | Acidotic breathing | | | | | | | | | | | Y | N | | E | |
|  |  |  |  |  |  |  |  |  |  |  |  |  |  |  |  |  |  |  |  |  |  |  |  |  |  | Wheeze | | | | | | | | | | | Y | N | | E | |
|  |  |  |  |  |  |  |  |  |  |  |  |  |  |  |  |  |  |  |  |  |  |  |  |  |  | Crackles | | | | | | | | | | | Y | N | | E | |
|  |  |  |  |  |  |  |  |  |  |  |  |  |  |  |  |  |  |  |  |  | Circulation | | | | | Pulse | | Weak | | | | | | Norm | | | E | /min | | | |
|  |  |  |  |  |  |  |  |  |  |  |  |  |  |  |  |  |  |  |  |  |  | | | | | Cap Refill | | | | | | | | X | | <2 | 2-3 | >3 | | E | |
|  |  |  |  |  |  |  |  |  |  |  |  |  |  |  |  |  |  |  |  |  |  |  |  |  |  | Skin temp | | Not cold | | | | | | hand | | | fore-arm | elbow | | E | |
|  |  |  |  |  |  |  |  |  |  |  |  |  |  |  |  |  |  |  |  |  |  |  |  |  |  | Pallor / Anaemia | | | | | | | | | | 0 | + | +++ | | E | |
| Diarrhoea | | | | Y | | | | |  | | | | E | | | |  | | | | Dehydration | | | | | Sunken eyes | | | | | | | | | | | Y | N | | E | |
| Diarrhoea > 14d | | | | Y | | | | | N | | | | E | | | |  |  |  |  |  | | | | | Skin pinch (sec) | | | | | | | | | | 0 | 1 | 2 | | E | |
| Diarrhoea bloody | | | | Y | | | | | N | | | | E | | | |  |  |  |  | Disability | | | | | AVPU | | | | | | | | | A | V | P | U | | E | |
| Convulsions | | | | Y | | | | | N | | | | E | | | |  |  |  |  |  | | | | | Can drink / breastfeed? | | | | | | | | | | | Y | N | | E | |
| If yes, no of fits | | | |  | | | | | | | | | | | | |  | | | |  | | | | | Bulging fontanelle | | | | | | | | | | | Y | N | | E | |
| Difficulty feeding | | | | Y | | | | | N | | | | E | | | |  | | | |  | | | | | Visible severe wasting | | | | | | | | | | | Y | N | | E | |
|  | | | | | | | | | | | | | | | | |  | | | |  | | | | | Oedema | | | | | | | none | | | foot | knee | face | | E | |
| Admission Diagnoses | | | | | | | | | | | | | | | | | | | | | | | | | | | | | | | | | | | | | | | | | |
| Malaria | | | □ Severe □ Non-sev □ No classif’n | | | | | | | | | | | | | | | | | | | | | | | | | | | Anaemia | | | | | | | □ Sev □ Non-sev | | | | |
| Pneumonia | | | □ V. Sev □ Sev □ Non-sev | | | | | | | | | | | | | | | | | | | | | | | | | | | Meningitis | | | | | | |  | | | | |
| Diarrhoea | | | □ Non-bloody □ Bloody | | | | | | | | | | | | | | | | | | | | | | | | | | |  | | | | | | |  | | | | |
| Dehydration | | | □ Shock □ Sev □ Some □ No classif’n | | | | | | | | | | | | | | | | | | | | | | | | | | |  | | | | | | |  | | | | |
| *HIV / AIDS* | | | □ previous diagnosis □ Clinical suspicion | | | | | | | | | | | | | | | | | | | | | | | | | | |  | | | | | | |  | | | | |
| *Malnutrition* | | | □ Kwash □ Marasm □ M. Kwash | | | | | | | | | | | | | | | | | | | | | | | | | | |  | | | | | | |  | | | | |
| *Other 1* | | |  | | | | | | | | | | | | | | | | | | | | | | | | | | | | | | | | | | | | | | |
| Investigations ordered | | | | | | | | | | | | | | | | | | | | | | | | | | | | | | | | | | | | | | | | | |
|  | | | Ordered? | | | | | | | | | | | | | | | | Results documented same day | | | | | | | | | | | Result (give units) | | | | | | | | | | | |
| Malaria Slide | | | Y / N | | | | | | | | | | | | | | | | Y / N / E | | | | | | | | | | | Pos / Neg | | | | | | | | | | | |
| Hb / HCT / PCV | | | Y / N | | | | | | | | | | | | | | | | Y / N / E | | | | | | | | | | |  | | | | | | | | | | | |
| HIV test | | | Y / N | | | | | | | | | | | | | | | | Y / N / E | | | | | | | | | | | Pos / Neg | | | | | | | | | | | |
| Glucose | | | Y / N | | | | | | | | | | | | | | | | Y / N / E | | | | | | | | | | |  | | | | | | | | | | | |
| Lumbar Puncture | | | Y / N | | | | | | | | | | | | | | | | Y / N / E (microscopy) | | | | | | | | | | | Pos / Neg | | | | | | | | | | | |
| HIV test (inpatient) | | | Y / N | | | | | | | | | | | | | | | | Y / N / E | | | | | | | | | | | Pos / Neg | | | | | | | | | | | |
| Other tests | | |  | | | | | | | | | | | | | | | |  | | | | | | | | | | |  | | | | | | | | | | | |
| Oxygen and Blood Transfusion – *Record data only about the immediate admission events* | | | | | | | | | | | | | | | | | | | | | | | | | | | | | | | | | | | | | | | | | |
|  | | | Ordered? | | | | | | | | | | | Describe how prescribed (flow rate, device) | | | | | | | | | | | | | | | | | | | | | | | | | | | |
| Oxygen | | | Y / N | | | | | | | | | | | □ No detail | | | | | | | | | Flow rate = | | | | | | | Ncath / NP / mask / Oth | | | | | | | | | | | |
| Transfusion | | | Y / N | | | | | | | | | | | □ No detail | | | | | | | | | Volume of Blood (mls) = | | | | | | | Duration prescribed (hrs) | | | | | | | | | | | |
| Treatment – *Record only the initial treatment prescribed for the admission episode* | | | | | | | | | | | | | | | | | | | | | | | | | | | | | | | | | | | | | | | | | |
|  | | | | Was drug prescribed? | | | | | | | | | | | | | | | Drug prescription | | | | | | | | | | | | | | | | | | | | | | |
|  | | | |  |  |  |  |  |  |  |  |  |  |  |  |  |  |  | Route | | | | Dose | | | | | | | Units | | | | | | | Freq | Days | | | |
| Antibiotics | | | | | | | | | | | | | | | | | | | | | | | | | | | | | | | | | | | | | | | | | |
| Penicillin | | | | Yes / No | | | | | | | | | | | | | | | iv / im | | | |  | | | | | | | mg / iu | | | | | | |  |  | | | |
| Gentamicin | | | | Yes / No | | | | | | | | | | | | | | | iv / im | | | |  | | | | | | | mg | | | | | | |  |  | | | |
| Amoxicillin | | | | Yes / No | | | | | | | | | | | | | | | po | | | |  | | | | | | | mg / mls / tabs | | | | | | |  |  | | | |
| ceftriaxone | | | | Yes / No | | | | | | | | | | | | | | | iv / im | | | |  | | | | | | | mg / mls / tabs | | | | | | |  |  | | | |
| chloramphenical | | | | Yes / No | | | | | | | | | | | | | | | iv / im | | | |  | | | | | | |  | | | | | | |  |  | | | |
| Metronidazole | | | | Yes / No | | | | | | | | | | | | | | | iv / po | | | |  | | | | | | | mg / mls / tabs | | | | | | |  |  | | | |
| Antimalarials | | | | | | | | | | | | | | | | | | | | | | | | | | | | | | | | | | | | | | | | | |
| Quinine (Load) | | | | | | | Yes / No | | | | | | | | | | | | iv / im | | | | |  | | | | | | mg | | | | | | | stat |  | | | |
| Quinine (Maint) | | | | | | | Yes / No | | | | | | | | | | | | iv / im / po | | | | |  | | | | | | mg | | | | | | |  |  | | | |
| Artemether - load | | | | | | | Yes / No | | | | | | | | | | | | iv / im | | | | |  | | | | | | mg | | | | | | | stat |  | | | |
| Artemether - maint | | | | | | | Yes / No | | | | | | | | | | | | iv / im | | | | |  | | | | | | mg | | | | | | |  |  | | | |
| Coartem | | | | | | | Yes / No | | | | | | | | | | | | po | | | | |  | | | | | | tabs | | | | | | |  |  | | | |
| Supportive Care | | | | | | | | | | | | | | | | | | | | | | | | | | | | | | | | | | | | | | | | | |
| Paracetamol | | Yes / No | | | | | | | | | | | | | | | | | im / po | | | | |  | | | | | | mg / mls / tabs | | | | | | |  |  | | | |
| Others | |  | | | | | | | | | | | | | | | | |  | | | | |  | | | | | |  | | | | | | |  |  | | | |
|  | |  | | | | | | | | | | | | | | | | |  | | | | |  | | | | | |  | | | | | | |  |  | | | |
| Did the child have a prescription for fluids to treat dehydration. | | | | | | | | | | | | | | | | | | | | | | | | | | | | | | Yes / No | | | | | | |  | | | | |
| A. Was the child given fluids using iv route? | | | | | | | | | | | | | | | | | | | | | | | | | Yes / No | | | | | *If No, skip to part B, if yes continue* | | | | | | | | | | | |
| Fluid prescribed | | | | | | | | | | | | | | | Fluid prescription for Step 1 and 2 (up to 6 hours) | | | | | | | | | | | | | | | | | | | | | | | | | | |
|  |  |  |  |  |  |  |  |  |  |  |  |  |  |  | Step 1 / Step 2 plan used | | | | | | | | | | Total Vol. | | | | | Time (hrs) | | | | | | |  | | | | |
| Ring / NSal / HS Darr / Other | | | | | | | | | | | | | | | Yes / No | | | | | | | | | |  | | | | |  | | | | | | |  | | | | |
| B. Was the child given fluids using oral or ng route? | | | | | | | | | | | | | | | | | | | | | | | | | Yes / No | | | | | *If No, then skip this section, if yes continue.* | | | | | | | | | | | |
| Fluid prescribed | | | | | | | | | | | | | | | Fluid prescription for first 4 hours | | | | | | | | | | | | | | | | | | | | | | | | | | |
|  |  |  |  |  |  |  |  |  |  |  |  |  |  |  | Ng tube used | | | | | | | | | | Total  Volume | | | | | Time  (hrs) | | | | | | | OR Volume with each stool | | | | |
| ORS / Ring / NSal / HSD / Other | | | | | | | | | | | | | | | Yes / No | | | | | | | | | |  | | | | |  | | | | | | | Mls/Not indicated | | | | |
| Was the child prescribed any feeds? | | | | | | | | | | | | | | | | | | | | | | | | | Yes / No | | | | | *If No, then skip this section, if yes continue.* | | | | | | | | | | | |
| Feed prescribed | | | | | | | | | | | | | | | Feed prescription for first 24 hours | | | | | | | | | | | | | | | | | | | | | | | | | | |
|  |  |  |  |  |  |  |  |  |  |  |  |  |  |  | Route | | | | | | | | | | | | | | | Feed vol | | | | | | | Freq / 24hrs | | | | |
| F75 / F100 / Sp Milk / HPD / Other | | | | | | | | | | | | | | | ngt / po | | | | | | | | | | | | | | | mls / E | | | | | | | / E | | | | |
| Fluid and Feed Monitoring | | | | | | | | | | | | | | | | | | | | | | | | | | | | | |  | | | | | | |  | | | | |
| Is there a feed/fluid monitoring chart for the first 24 hrs | | | | | | | | | | | | | | | | | | | | | | | | | | | | | | Yes / No | | | | | | | | | | | |
| Were feeds/fluids monitored as prescribed for the first 24 hrs | | | | | | | | | | | | | | | | | | | | | | | | | | | | | | Yes / No | | | | | | | | | | | |
| Ward rounds | | | | | | | | | | | | | | | | | | | | | | | | | | | | | |  | | | | | | | | | | | |
| No of documented ward rounds | | | | | | | | | | | | | | | | | | | | | | | | | | | | | |  | | | | | | | | | | | |
| No of documented major (consultant) ward rounds | | | | | | | | | | | | | | | | | | | | | | | | | | | | | |  | | | | | | | | | | | |
| Vital signs chart | | | | | | | | | | | | | | | | | | | | | | | | | | | | | | | | | | | | | | | | | |
| Is there a vital signs chart? | | | | | | | | | | | | | | | | | | | | | | | | | | | | | | | | | | | | | Y □ N□ | | | | |
| What parameters are recorded? | | | | | | | | | | Temperature | | | | | | | | | | | | Respiratory rate | | | | | | | | Pulse rate | | | | | | | Oxygen saturation | | | | |
| No of times documented in 48 hrs. | | | | | | | | | |  | | | | | | | | | | | |  | | | | | | | |  | | | | | | |  | | | | |
| Is there a discharge/Death summary in the case record? Y □ N □ | | | | | | | | | | | | | | | | | | | | | | | | | | | | | | | | | | | | | | | | | |
| Discharge Date | | | | | / /2001 | | | | | | | | | | | | | Outcome | | | | | | | | | | | | Alive / Dead / Refer’d / Absc’d | | | | | | | | | | | |
| Discharge Diagnoses: Select ONE *primary diagnosis (tick 1) and secondary diagnoses (tick 2)* | | | | | | | | | | | | | | | | | | | | | | | | | | | | | | | | | | | | | | | | | |
| Is there a clear primary diagnosis | | | | | | | | | | | | | | | | | | | | | | Y □ N □ | | | | | | | | | | | | | | | | | | | |
| Malaria | | | | | 1 | | | 2 | | □ Severe □ Non-sev □ No classif’n | | | | | | | | | | | | | | | | | | | | Meningitis | | | | | | | 1 | | 2 | | |
| Pneumonia | | | | | 1 | | | 2 | | □ V. Sev □ Sev □ Non-sev | | | | | | | | | | | | | | | | | | | |  | | | | | | | | | | | |
| Diarrhoea | | | | | 1 | | | 2 | | □ Non-bloody □ Bloody | | | | | | | | | | | | | | | | | | | | *Other diagnosis (name)* | | | | | | | 1 | | 2 | | |
| Dehydration | | | | | 1 | | | 2 | | □ Sev □ Some □ No classif’n | | | | | | | | | | | | | | | | | | | |  | | | | | | | | | | | |
| *HIV / AIDS* | | | | | 1 | | | 2 | | □ previous □ clinical suspicion  diagnosis | | | | | | | | | | | | | | | | | | | |  | | | | | | | | | | | |
| Malnutrition | | | | | 1 | | | 2 | | □ Kwash □ Marasm □ M. Kwash | | | | | | | | | | | | | | | | | | | |  | | | | | | | | | | | |
| Anaemia | | | | | 1 | | | 2 | | □ Sev □ Non-sev | | | | | | | | | | | | | | | | | | | |  | | | | | | | | | | | |
| Last weight recorded | | | | |  | | | | | | | | | | | | | | | | | Date recorded | | | | | | | |  | | | | | | | | | | | |
| Follow Up | | | | | Not arranged | | | | | | | | | | | | | | | | | Hospital | | | | | | | | Disp / H. Centre | | | | | | | | | | | |
| Did the child have a diagnosis of malaria or meningitis? | | | | | | | | | | | | | | | | | | | | | | | | | | | | | | Yes / No | | | | | | |  | | | | |
| Were there convulsions | | | | | | | | | | | | | | | | | | | | | | Yes / No | | | | | | | | *If No, skip to part B, if yes continue* | | | | | | | | | | | |
|  | How many convulsions in 24 hrs | | | | | | | | | | | | | | | | | | | | | □ 1 -2 □ 3 -4 □>5 | | | | | | | | | | | | | | | | | | | |
|  | Type of convulsion experienced | | | | | | | | | | | | | | | | | | | | | □Generalized □ Focal □ Empty | | | | | | | | | | | | | | | | | | | |
| Was Lumber puncture done | | | | | | | | | | | | | | | | | | | | | | Yes / No | | | | | | | | *If No, then skip this section, if yes continue.* | | | | | | | | | | | |
|  | Bed side exam of CSF | | | | | | | | | | | | | | | | | | | | | □ dry tap □ under pressure  □ turbid □ bloody □ clear □ not done | | | | | | | | | | | | | | | | | | | |
|  |  | | | | | | | | | | | | | | | CSF investigations | | | | | | | | | | | | | | | | | | | | | | | | | |
|  |  | | | | | | | | | | | | | | | Test done | | | | | | | | | | | | | | Test results | | | | | | | | | | | |
|  | CSF microscopy | | | | | | | | | | | | | | | Yes / No | | | | | | | | | | | | | |  | | | | | | | | | | | |
|  | CSF Biochemistry | | | | | | | | | | | | | | | Yes / No | | | | | | | | | | | | | |  | | | | | | | | | | | |
|  | CSF culture | | | | | | | | | | | | | | | Yes / No | | | | | | | | | | | | | | □ no growth □ growth  (list)……………………………... | | | | | | | | | | | |
| What was the outcome? | | | | | | | | | | | | | | | | | | | | | | | | | | | | | | | | | | | | | | | | | |
| □ Died □ Alive no sequelae □ Alive with sequelae □ Referred □ Absconded □ empty | | | | | | | | | | | | | | | | | | | | | | | | | | | | | | | | | | | | | | | | | |
|  | If sequelae please give details | | | | | | | | | | | | | | | | | | | | | | | | | | | | | | | | | | | | | | | | |
|  |  | | | | | | | | | | | | | | | | | | | | | | | | | | | | | | | | | | | | | | | | |
