# Supplementary figures and images for: The Paediatric Admission Quality of Care (PAQC) score: designing a tool to measure the quality of early inpatient paediatric care in a low‐income setting
Source: Trop Med Int Health. 2016 Aug 10;21(10):1334–45. doi: 10.1111/tmi.12752 (PMC5053245; doi:10.1111/tmi.12752)

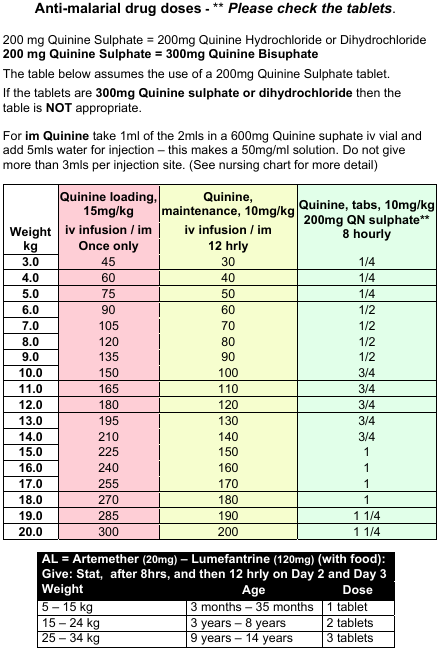

Supplement: Supplementary file 2 — Appendix S2. Anti‐malarial drug doses. [file TMI-21-1334-s002.png]

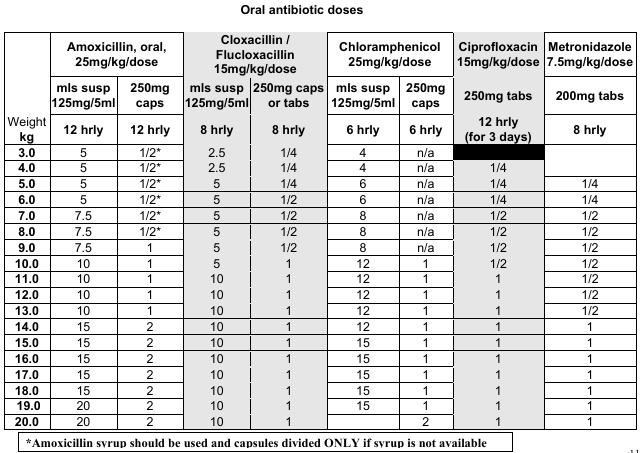

Supplement: Supplementary file 3 — Appendix S3. Oral antibiotic doses. [file TMI-21-1334-s003.png]

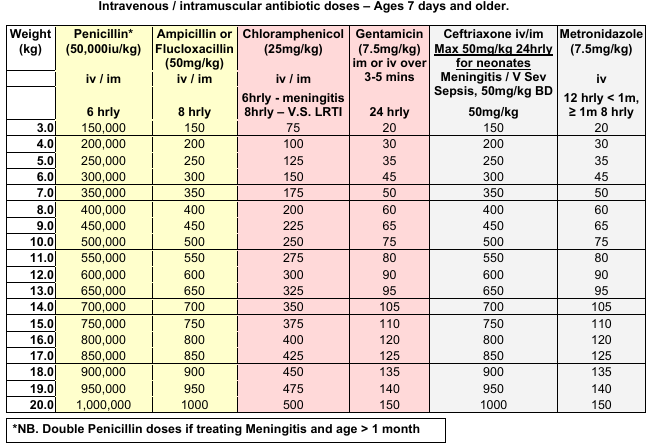

Supplement: Supplementary file 4 — Appendix S4. Intravenous/intramuscular antibiotic doses – ages 7 days and older. [file TMI-21-1334-s004.png]

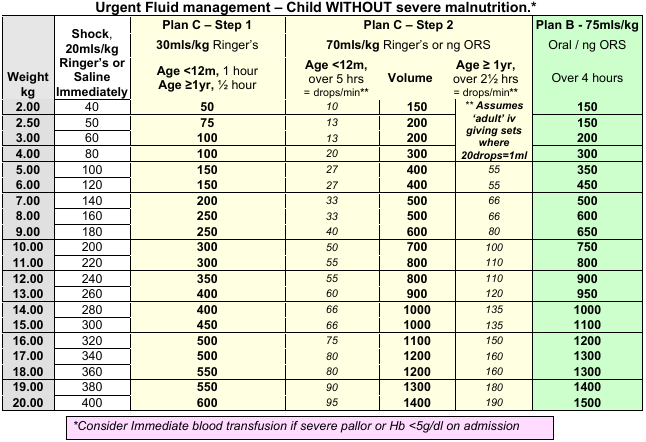

Supplement: Supplementary file 5 — Appendix S5. Urgent fluid management – child without severe malnutrition. [file TMI-21-1334-s005.png]
