# Supplementary material for: The Paediatric Admission Quality of Care (PAQC) score: designing a tool to measure the quality of early inpatient paediatric care in a low‐income setting
Source: Trop Med Int Health. 2016 Aug 10;21(10):1334–45. doi: 10.1111/tmi.12752 (PMC5053245; doi:10.1111/tmi.12752)
Supplement: Supplementary file 6 — Appendix S6. Table S1. Number of episodes of each disease/multi‐morbidity across surveys. Table S2. Tetrachoric correlation matrix of assessment items in the basic score. Table S3. Tetrachoric correlation matrix of treatment items in the basic score. Table S4. Tetrachoric correlation matrix of items in the malaria, pneumonia and diarrhoea/dehydration component PAQC scores. [file TMI-21-1334-s006.docx]

| Supplementary Table 1: Number of episodes of each disease/multi-morbidity across surveys | | | | | | | |
| --- | --- | --- | --- | --- | --- | --- | --- |
|  | **Survey** | | | | | | |
| **Disease** | Baseline | 1^st^ follow-up | 2^nd^ follow-up | End-point | 1^st^ post-intervention | 2^nd^ post-intervention | **Total** |
| None* | 262 | 233 | 254 | 234 | 122 | 147 | **1,252** |
| Malaria | 953 | 646 | 581 | 750 | 312 | 437 | **3,679** |
| Pneumonia | 304 | 336 | 350 | 419 | 157 | 173 | **1,739** |
| Diarrhoea** | 108 | 102 | 111 | 208 | 99 | 104 | **732** |
| Malaria+Pneumonia | 548 | 494 | 600 | 579 | 333 | 269 | **2,823** |
| Malaria+Diarrhoea | 225 | 172 | 155 | 328 | 102 | 159 | **1,141** |
| Pneumonia+Diarrhoea | 13 | 55 | 35 | 64 | 25 | 31 | **223** |
| Malaria+Pneumonia+  Diarrhoea | 37 | 81 | 90 | 132 | 56 | 50 | **446** |
| **Total** | **2,450** | **2,119** | **2,176** | **2,714** | **1,206** | **1,370** | **12,035†** |
| *May include other diseases than the three considered here; **Diarrhoea with or without dehydration at each mention; †One observation is missing a survey indicator | | | | | | | |

| Supplementary Table 2: Tetrachoric correlation matrix of assessment items in the basic score | | | | | | | | |
| --- | --- | --- | --- | --- | --- | --- | --- | --- |
| *Malaria* | **fever** | **convulsions** | **acidotic breathing** | **pallor** | **(in)ability to drink** | **level of consciousness** | **indrawing** | **malaria test** |
| **fever** | 1.00 |  |  |  |  |  |  |  |
| **convulsions** | 0.64 | 1.00 |  |  |  |  |  |  |
| **acidotic breathing** | 0.64 | 0.94 | 1.00 |  |  |  |  |  |
| **pallor** | 0.30 | 0.44 | 0.52 | 1.00 |  |  |  |  |
| **(in)ability to drink** | 0.62 | 0.91 | 0.99 | 0.52 | 1.00 |  |  |  |
| **level of consciousness** | 0.58 | 0.87 | 0.92 | 0.54 | 0.92 | 1.00 |  |  |
| **indrawing** | 0.61 | 0.89 | 0.99 | 0.49 | 0.97 | 0.90 | 1.00 |  |
| **malaria test** | 0.20 | 0.23 | 0.25 | 0.13 | 0.25 | 0.30 | 0.22 | 1.00 |
| *Pneumonia* | **cough** | **difficult breathing** | **central cyanosis** | **(in)ability to drink** | **level of consciousness** | **grunting** | **indrawing** | **resp. rate** |
| **cough** | 1.00 |  |  |  |  |  |  |  |
| **difficult breathing** | 0.55 | 1.00 |  |  |  |  |  |  |
| **central cyanosis** | 0.52 | 0.64 | 1.00 |  |  |  |  |  |
| **(in)ability to drink** | 0.68 | 0.79 | 0.89 | 1.00 |  |  |  |  |
| **level of consciousness** | 0.58 | 0.71 | 0.82 | 0.90 | 1.00 |  |  |  |
| **grunting** | 0.65 | 0.80 | 0.92 | 0.98 | 0.91 | 1.00 |  |  |
| **indrawing** | 0.59 | 0.78 | 0.86 | 0.95 | 0.87 | 0.97 | 1.00 |  |
| **resp. rate** | 0.39 | 0.38 | 0.45 | 0.59 | 0.54 | 0.59 | 0.58 | 1.00 |
| *Diarrhoea/ dehydration* | **diarrhoea** | **vomiting** | **capillary refill** | **level of consciousness** | **(in)ability to drink** | **sunken eyes** | **skin pinch** | **pulse** |
| **diarrhoea** | 1.00 |  |  |  |  |  |  |  |
| **vomiting** | 0.61 | 1.00 |  |  |  |  |  |  |
| **capillary refill** | 0.36 | 0.91 | 1.00 |  |  |  |  |  |
| **level of consciousness** | 0.37 | 0.84 | 0.86 | 1.00 |  |  |  |  |
| **(in)ability to drink** | 0.42 | 0.94 | 0.93 | 0.88 | 1.00 |  |  |  |
| **sunken eyes** | 0.46 | 0.91 | 0.94 | 0.88 | 0.97 | 1.00 |  |  |
| **indrawing** | 0.42 | 0.91 | 0.94 | 0.87 | 0.95 | 0.97 | 1.00 |  |
| **pulse** | 0.41 | 0.80 | 0.79 | 0.74 | 0.83 | 0.84 | 0.82 | 1.00 |

| Supplementary Table 3: Tetrachoric correlation matrix of treatment items in the basic score | | | | | | | | | | | | | | | | | | | | |
| --- | --- | --- | --- | --- | --- | --- | --- | --- | --- | --- | --- | --- | --- | --- | --- | --- | --- | --- | --- | --- |
|  |  | **Malaria** | | | | | **Pneumonia** | | | | | | | **Diarrhoea/**  **Dehydration** | | | | | | |
|  |  | **drug** | **route** | **dose** | **freq.** | **dur.** | **drug** | **route** | | **dose** | **freq.** | | **dur.** | | | **drug** | | **dose** | | **freq.** |
| **Malaria** | **drug** | 1.00 |  |  |  |  |  |  | |  |  | |  | | |  | |  | |  |
|  | **route** | 0.80 | 1.00 |  |  |  |  |  | |  |  | |  | | |  | |  | |  |
|  | **dose** | 0.26 | 0.64 | 1.00 |  |  |  |  | |  |  | |  | | |  | |  | |  |
|  | **freq.** | 0.85 | 0.93 | 0.75 | 1.00 |  |  |  | |  |  | |  | | |  | |  | |  |
|  | **dur.** | 0.12 | 0.27 | 0.91 | 0.58 | 1.00 |  |  | |  |  | |  | | |  | |  | |  |
| **Pneumonia** | **drug** |  |  |  |  |  | 1.00 |  | |  |  | |  | | |  | |  | |  |
|  | **route** |  |  |  |  |  | 0.75 | 1.00 | |  |  | |  | | |  | |  | |  |
|  | **dose** |  |  |  |  |  | 0.33 | 0.85 | | 1.00 |  | |  | | |  | |  | |  |
|  | **freq.** |  |  |  |  |  | 0.68 | 0.97 | | 0.90 | 1.00 | |  | | |  | |  | |  |
|  | **dur.** |  |  |  |  |  | 0.65 | 0.93 | | 0.81 | 0.94 | | 1.00 | | |  | |  | |  |
| **DnD** | **drug** |  |  |  |  |  |  | |  |  |  |  | | | 1.00 | |  | |  | |
|  | **dose** |  |  |  |  |  |  | |  |  |  |  | | | 0.66 | | 1.00 | |  | |
|  | **freq.** |  |  |  |  |  |  | |  |  |  |  | | | 0.44 | | 0.19 | | 1.00 | |

| Supplementary Table 4: Tetrachoric correlation matrix of items in the malaria, pneumonia and diarrhoea/dehydration component PAQC scores | | | | | | | | | | | | | | | | |
| --- | --- | --- | --- | --- | --- | --- | --- | --- | --- | --- | --- | --- | --- | --- | --- | --- |
|  |  | **Malaria** | | | | | **Pneumonia** | | | | | **Diarrhoea/Dehydration** | | | | |
|  |  | **pri.** | **sec.** | **class.** | **drug** | **use** | **pri.** | **sec.** | **class.** | **drug** | **use** | **pri.** | **sec.** | **class.** | **drug** | **use** |
| **Malaria** | **pri.** | 1.00 |  |  |  |  |  |  |  |  |  |  |  |  |  |  |
|  | **sec.** | 0.60 | 1.00 |  |  |  |  |  |  |  |  |  |  |  |  |  |
|  | **class.** | 0.56 | 0.83 | 1.00 |  |  |  |  |  |  |  |  |  |  |  |  |
|  | **drug** | 0.46 | 0.66 | 1.00 | 1.00 |  |  |  |  |  |  |  |  |  |  |  |
|  | **use** | 0.24 | 0.49 | 0.48 | 0.45 | 1.00 |  |  |  |  |  |  |  |  |  |  |
| **Pneumonia** | **pri.** |  |  |  |  |  | 1.00 |  |  |  |  |  |  |  |  |  |
|  | **sec.** |  |  |  |  |  | 0.62 | 1.00 |  |  |  |  |  |  |  |  |
|  | **class.** |  |  |  |  |  | 0.49 | 1.00 | 1.00 |  |  |  |  |  |  |  |
|  | **drug** |  |  |  |  |  | 0.23 | 0.57 | 1.00 | 1.00 |  |  |  |  |  |  |
|  | **use** |  |  |  |  |  | 0.15 | 0.28 | 0.28 | 0.51 | 1.00 |  |  |  |  |  |
| **DnD** | **pri.** |  |  |  |  |  |  |  |  |  |  | 1.00 |  |  |  |  |
|  | **sec.** |  |  |  |  |  |  |  |  |  |  | 0.34 | 1.00 |  |  |  |
|  | **class.** |  |  |  |  |  |  |  |  |  |  | 0.15 | 0.87 | 1.00 |  |  |
|  | **drug** |  |  |  |  |  |  |  |  |  |  | 0.11 | 0.24 | 0.27 | 1.00 |  |
|  | **use** |  |  |  |  |  |  |  |  |  |  | 0.21 | 0.24 | 0.26 | 0.60 | 1.00 |
